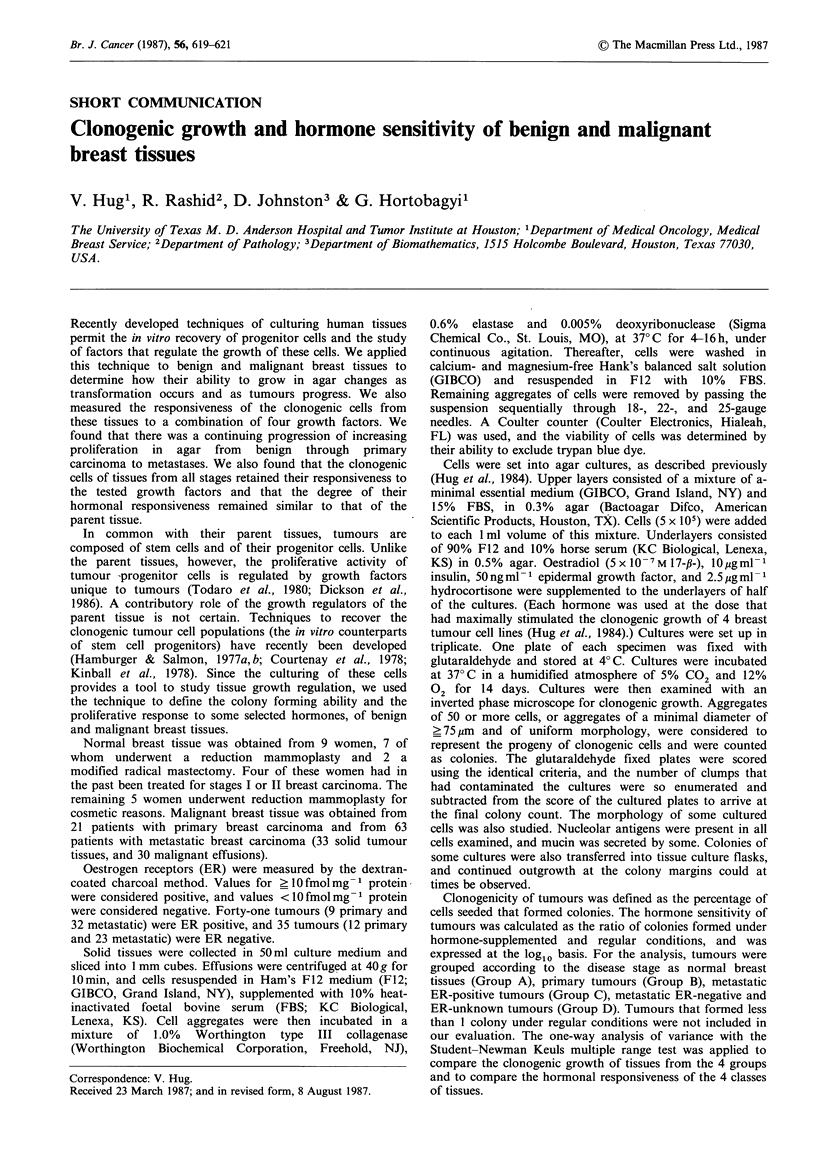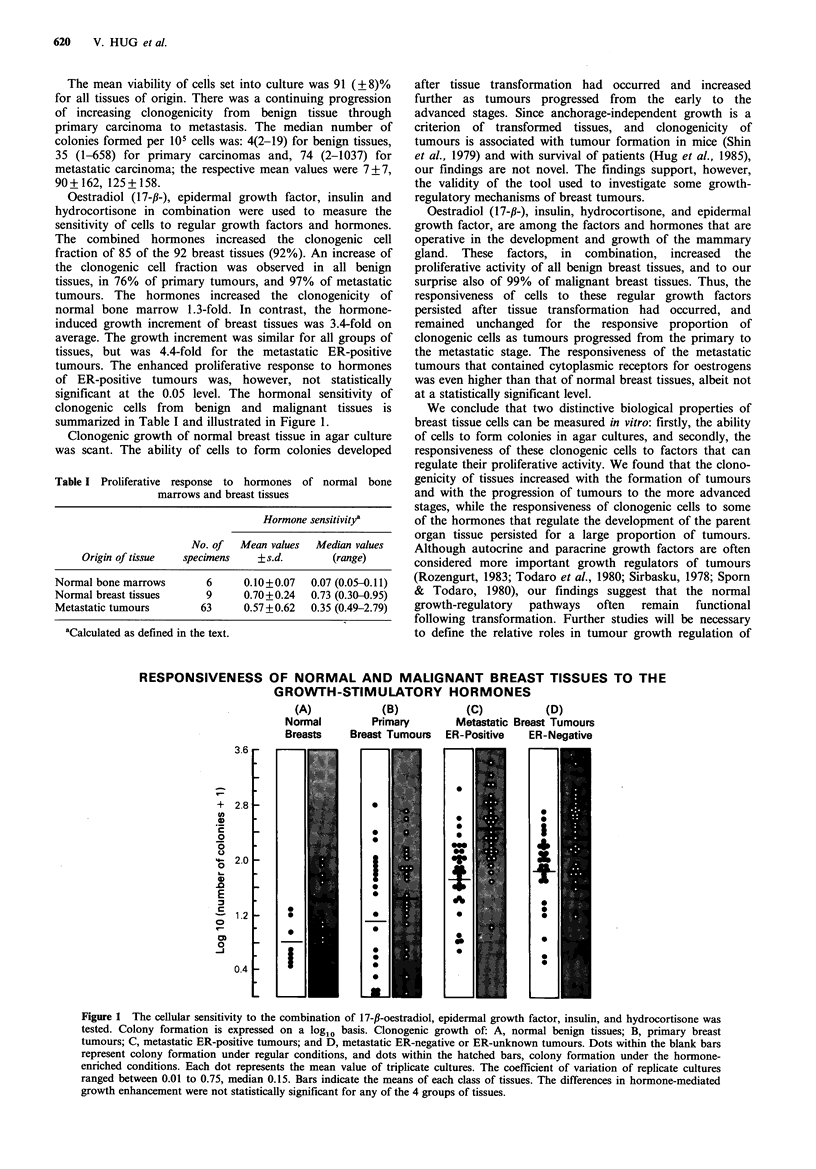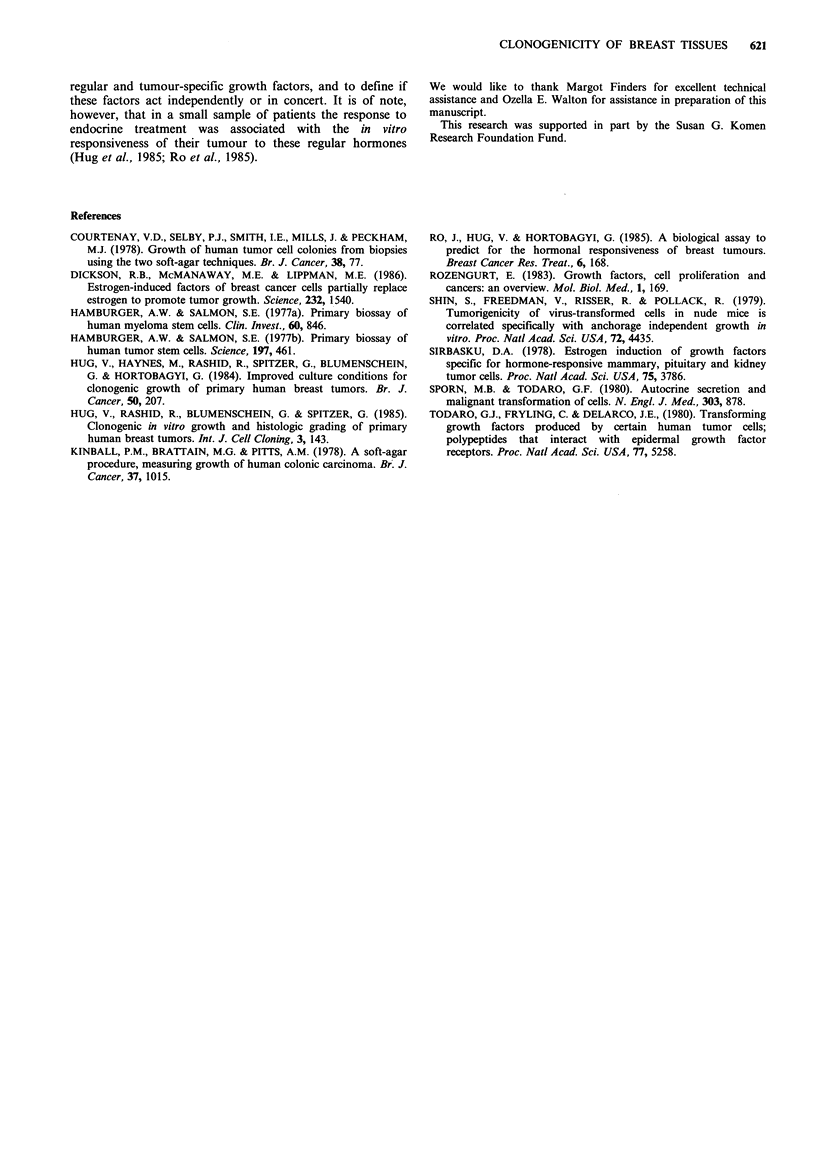# Clonogenic growth and hormone sensitivity of benign and malignant breast tissues.

**DOI:** 10.1038/bjc.1987.253

**Published:** 1987-11

**Authors:** V. Hug, R. Rashid, D. Johnston, G. Hortobagyi

**Affiliations:** University of Texas M. D. Anderson Hospital and Tumor Institute, Department of Medical Oncology, Houston 77030.


*Br. J. Cancer* (1987), **56**, 619–621 © The Macmillan Press Ltd., 1987

SHORT COMMUNICATION

### Clonogenic growth and hormone sensitivity of benign and malignant breast tissues

V. Hug[1], R. Rashid[2], D. Johnston[3] & G. Hortobagyi[1]

*The University of Texas M. D. Anderson Hospital and Tumor Institute at Houston; [1]Department of Medical Oncology, Medical Breast Service; [2]Department of Pathology; [3]Department of Biomathematics, 1515 Holcombe Boulevard, Houston, Texas 77030, USA.*

Recently developed techniques of culturing human tissues permit the *in vitro* recovery of progenitor cells and the study of factors that regulate the growth of these cells. We applied this technique to benign and malignant breast tissues to determine how their ability to grow in agar changes as transformation occurs and as tumours progress. We also measured the responsiveness of the clonogenic cells from these tissues to a combination of four growth factors. We found that there was a continuing progression of increasing proliferation in agar from benign through primary carcinoma to metastases. We also found that the clonogenic cells of tissues from all stages retained their responsiveness to the tested growth factors and that the degree of their hormonal responsiveness remained similar to that of the parent tissue.

In common with their parent tissues, tumours are composed of stem cells and of their progenitor cells. Unlike the parent tissues, however, the proliferative activity of tumour progenitor cells is regulated by growth factors unique to tumours (Todaro *et al.*, 1980; Dickson *et al.*, 1986). A contributory role of the growth regulators of the parent tissue is not certain. Techniques to recover the clonogenic tumour cell populations (the *in vitro* counterparts of stem cell progenitors) have recently been developed (Hamburger & Salmon, 1977*a,b*; Courtenay *et al.*, 1978; Kinball *et al.*, 1978). Since the culturing of these cells provides a tool to study tissue growth regulation, we used the technique to define the colony forming ability and the proliferative response to some selected hormones, of benign and malignant breast tissues.

Normal breast tissue was obtained from 9 women, 7 of whom underwent a reduction mammoplasty and 2 a modified radical mastectomy. Four of these women had in the past been treated for stages I or II breast carcinoma. The remaining 5 women underwent reduction mammoplasty for cosmetic reasons. Malignant breast tissue was obtained from 21 patients with primary breast carcinoma and from 63 patients with metastatic breast carcinoma (33 solid tumour tissues, and 30 malignant effusions).

Oestrogen receptors (ER) were measured by the dextran-coated charcoal method. Values for $\geqq 10\,fmol\,mg^{-1}$ protein were considered positive, and values $< 10\,fmol\,mg^{-1}$ protein were considered negative. Forty-one tumours (9 primary and 32 metastatic) were ER positive, and 35 tumours (12 primary and 23 metastatic) were ER negative.

Solid tissues were collected in 50 ml culture medium and sliced into 1 mm cubes. Effusions were centrifuged at 40 *g* for 10 min, and cells resuspended in Ham's F12 medium (F12; GIBCO, Grand Island, NY), supplemented with 10% heat-inactivated foetal bovine serum (FBS; KC Biological, Lenexa, KS). Cell aggregates were then incubated in a mixture of 1.0% Worthington type III collagenase (Worthington Biochemical Corporation, Freehold, NJ), 0.6% elastase and 0.005% deoxyribonuclease (Sigma Chemical Co., St. Louis, MO), at 37°C for 4–16 h, under continuous agitation. Thereafter, cells were washed in calcium- and magnesium-free Hank's balanced salt solution (GIBCO) and resuspended in F12 with 10% FBS. Remaining aggregates of cells were removed by passing the suspension sequentially through 18-, 22-, and 25-gauge needles. A Coulter counter (Coulter Electronics, Hialeah, FL) was used, and the viability of cells was determined by their ability to exclude trypan blue dye.

Cells were set into agar cultures, as described previously (Hug *et al.*, 1984). Upper layers consisted of a mixture of a-minimal essential medium (GIBCO, Grand Island, NY) and 15% FBS, in 0.3% agar (Bactoagar Difco, American Scientific Products, Houston, TX). Cells ($5\times10^5$) were added to each 1 ml volume of this mixture. Underlayers consisted of 90% F12 and 10% horse serum (KC Biological, Lenexa, KS) in 0.5% agar. Oestradiol ($5\times10^{-7}$ M 17-$\beta$-), $10\,\mu g\,ml^{-1}$ insulin, $50\,ng\,ml^{-1}$ epidermal growth factor, and $2.5\,\mu g\,ml^{-1}$ hydrocortisone were supplemented to the underlayers of half of the cultures. (Each hormone was used at the dose that had maximally stimulated the clonogenic growth of 4 breast tumour cell lines (Hug *et al.*, 1984).) Cultures were set up in triplicate. One plate of each specimen was fixed with glutaraldehyde and stored at 4°C. Cultures were incubated at 37°C in a humidified atmosphere of 5% $CO_2$ and 12% $O_2$ for 14 days. Cultures were then examined with an inverted phase microscope for clonogenic growth. Aggregates of 50 or more cells, or aggregates of a minimal diameter of $\geqq 75\,\mu m$ and of uniform morphology, were considered to represent the progeny of clonogenic cells and were counted as colonies. The glutaraldehyde fixed plates were scored using the identical criteria, and the number of clumps that had contaminated the cultures were so enumerated and subtracted from the score of the cultured plates to arrive at the final colony count. The morphology of some cultured cells was also studied. Nucleolar antigens were present in all cells examined, and mucin was secreted by some. Colonies of some cultures were also transferred into tissue culture flasks, and continued outgrowth at the colony margins could at times be observed.

Clonogenicity of tumours was defined as the percentage of cells seeded that formed colonies. The hormone sensitivity of tumours was calculated as the ratio of colonies formed under hormone-supplemented and regular conditions, and was expressed at the $\log_{10}$ basis. For the analysis, tumours were grouped according to the disease stage as normal breast tissues (Group A), primary tumours (Group B), metastatic ER-positive tumours (Group C), metastatic ER-negative and ER-unknown tumours (Group D). Tumours that formed less than 1 colony under regular conditions were not included in our evaluation. The one-way analysis of variance with the Student–Newman Keuls multiple range test was applied to compare the clonogenic growth of tissues from the 4 groups and to compare the hormonal responsiveness of the 4 classes of tissues.

Correspondence: V. Hug.
Received 23 March 1987; and in revised form, 8 August 1987.

The mean viability of cells set into culture was 91 (±8)% for all tissues of origin. There was a continuing progression of increasing clonogenicity from benign tissue through primary carcinoma to metastasis. The median number of colonies formed per $10^5$ cells was: 4(2–19) for benign tissues, 35 (1–658) for primary carcinomas and, 74 (2–1037) for metastatic carcinoma; the respective mean values were $7 \pm 7$, $90 \pm 162$, $125 \pm 158$.

Oestradiol (17-β-), epidermal growth factor, insulin and hydrocortisone in combination were used to measure the sensitivity of cells to regular growth factors and hormones. The combined hormones increased the clonogenic cell fraction of 85 of the 92 breast tissues (92%). An increase of the clonogenic cell fraction was observed in all benign tissues, in 76% of primary tumours, and 97% of metastatic tumours. The hormones increased the clonogenicity of normal bone marrow 1.3-fold. In contrast, the hormone-induced growth increment of breast tissues was 3.4-fold on average. The growth increment was similar for all groups of tissues, but was 4.4-fold for the metastatic ER-positive tumours. The enhanced proliferative response to hormones of ER-positive tumours was, however, not statistically significant at the 0.05 level. The hormonal sensitivity of clonogenic cells from benign and malignant tissues is summarized in Table I and illustrated in Figure 1.

Clonogenic growth of normal breast tissue in agar culture was scant. The ability of cells to form colonies developed after tissue transformation had occurred and increased further as tumours progressed from the early to the advanced stages. Since anchorage-independent growth is a criterion of transformed tissues, and clonogenicity of tumours is associated with tumour formation in mice (Shin *et al.*, 1979) and with survival of patients (Hug *et al.*, 1985), our findings are not novel. The findings support, however, the validity of the tool used to investigate some growth-regulatory mechanisms of breast tumours.

**Table I** Proliferative response to hormones of normal bone marrows and breast tissues

| | | *Hormone sensitivity*[a] | |
|---|---|---|---|
| *Origin of tissue* | *No. of specimens* | *Mean values ±s.d.* | *Median values (range)* |
| Normal bone marrows | 6 | 0.10±0.07 | 0.07 (0.05–0.11) |
| Normal breast tissues | 9 | 0.70±0.24 | 0.73 (0.30–0.95) |
| Metastatic tumours | 63 | 0.57±0.62 | 0.35 (0.49–2.79) |

[a]Calculated as defined in the text.

Oestradiol (17-β-), insulin, hydrocortisone, and epidermal growth factor, are among the factors and hormones that are operative in the development and growth of the mammary gland. These factors, in combination, increased the proliferative activity of all benign breast tissues, and to our surprise also of 99% of malignant breast tissues. Thus, the responsiveness of cells to these regular growth factors persisted after tissue transformation had occurred, and remained unchanged for the responsive proportion of clonogenic cells as tumours progressed from the primary to the metastatic stage. The responsiveness of the metastatic tumours that contained cytoplasmic receptors for oestrogens was even higher than that of normal breast tissues, albeit not at a statistically significant level.

We conclude that two distinctive biological properties of breast tissue cells can be measured *in vitro*: firstly, the ability of cells to form colonies in agar cultures, and secondly, the responsiveness of these clonogenic cells to factors that can regulate their proliferative activity. We found that the clonogenicity of tissues increased with the formation of tumours and with the progression of tumours to the more advanced stages, while the responsiveness of clonogenic cells to some of the hormones that regulate the development of the parent organ tissue persisted for a large proportion of tumours. Although autocrine and paracrine growth factors are often considered more important growth regulators of tumours (Rozengurt, 1983; Todaro *et al.*, 1980; Sirbasku, 1978; Sporn & Todaro, 1980), our findings suggest that the normal growth-regulatory pathways often remain functional following transformation. Further studies will be necessary to define the relative roles in tumour growth regulation of

**RESPONSIVENESS OF NORMAL AND MALIGNANT BREAST TISSUES TO THE GROWTH-STIMULATORY HORMONES**

**Figure 1** The cellular sensitivity to the combination of 17-β-oestradiol, epidermal growth factor, insulin, and hydrocortisone was tested. Colony formation is expressed on a $\log_{10}$ basis. Clonogenic growth of: A, normal benign tissues; B, primary breast tumours; C, metastatic ER-positive tumours; and D, metastatic ER-negative or ER-unknown tumours. Dots within the blank bars represent colony formation under regular conditions, and dots within the hatched bars, colony formation under the hormone-enriched conditions. Each dot represents the mean value of triplicate cultures. The coefficient of variation of replicate cultures ranged between 0.01 to 0.75, median 0.15. Bars indicate the means of each class of tissues. The differences in hormone-mediated growth enhancement were not statistically significant for any of the 4 groups of tissues.

regular and tumour-specific growth factors, and to define if these factors act independently or in concert. It is of note, however, that in a small sample of patients the response to endocrine treatment was associated with the *in vitro* responsiveness of their tumour to these regular hormones (Hug *et al.*, 1985; Ro *et al.*, 1985).

We would like to thank Margot Finders for excellent technical assistance and Ozella E. Walton for assistance in preparation of this manuscript.

This research was supported in part by the Susan G. Komen Research Foundation Fund.

**References**

COURTENAY, V.D., SELBY, P.J., SMITH, I.E., MILLS, J. & PECKHAM, M.J. (1978). Growth of human tumor cell colonies from biopsies using the two soft-agar techniques. *Br. J. Cancer*, **38**, 77.

DICKSON, R.B., McMANAWAY, M.E. & LIPPMAN, M.E. (1986). Estrogen-induced factors of breast cancer cells partially replace estrogen to promote tumor growth. *Science*, **232**, 1540.

HAMBURGER, A.W. & SALMON, S.E. (1977a). Primary biossay of human myeloma stem cells. *Clin. Invest.*, **60**, 846.

HAMBURGER, A.W. & SALMON, S.E. (1977b). Primary biossay of human tumor stem cells. *Science*, **197**, 461.

HUG, V., HAYNES, M., RASHID, R., SPITZER, G., BLUMENSCHEIN, G. & HORTOBAGYI, G. (1984). Improved culture conditions for clonogenic growth of primary human breast tumors. *Br. J. Cancer*, **50**, 207.

HUG, V., RASHID, R., BLUMENSCHEIN, G. & SPITZER, G. (1985). Clonogenic *in vitro* growth and histologic grading of primary human breast tumors. *Int. J. Cell Cloning*, **3**, 143.

KINBALL, P.M., BRATTAIN, M.G. & PITTS, A.M. (1978). A soft-agar procedure, measuring growth of human colonic carcinoma. *Br. J. Cancer*, **37**, 1015.

RO, J., HUG, V. & HORTOBAGYI, G. (1985). A biological assay to predict for the hormonal responsiveness of breast tumours. *Breast Cancer Res. Treat.*, **6**, 168.

ROZENGURT, E. (1983). Growth factors, cell proliferation and cancers: an overview. *Mol. Biol. Med.*, **1**, 169.

SHIN, S., FREEDMAN, V., RISSER, R. & POLLACK, R. (1979). Tumorigenicity of virus-transformed cells in nude mice is correlated specifically with anchorage independent growth *in vitro*. *Proc. Natl Acad. Sci. USA*, **72**, 4435.

SIRBASKU, D.A. (1978). Estrogen induction of growth factors specific for hormone-responsive mammary, pituitary and kidney tumor cells. *Proc. Natl Acad. Sci. USA*, **75**, 3786.

SPORN, M.B. & TODARO, G.F. (1980). Autocrine secretion and malignant transformation of cells. *N. Engl. J. Med.*, **303**, 878.

TODARO, G.J., FRYLING, C. & DELARCO, J.E., (1980). Transforming growth factors produced by certain human tumor cells; polypeptides that interact with epidermal growth factor receptors. *Proc. Natl Acad. Sci. USA*, **77**, 5258.